# Editorial: The Neurophysiology of Developmental Stuttering: Unraveling the Mysteries of Fluency

**DOI:** 10.3389/fnhum.2021.833870

**Published:** 2022-01-27

**Authors:** Pierpaolo Busan, Nicole E. Neef, Maja Rogić Vidaković, Piero Paolo Battaglini, Martin Sommer

**Affiliations:** ^1^IRCCS Ospedale San Camillo, Venice, Italy; ^2^Department of Diagnostic and Interventional Neuroradiology, University Medical Center Göttingen, Göttingen, Germany; ^3^University of Split, School of Medicine, Department of Neuroscience, Laboratory for Human and Experimental Neurophysiology, Split, Croatia; ^4^Department of Life Sciences, University of Trieste, Trieste, Italy; ^5^Department of Neurology, University Medical Center Göttingen, Göttingen, Germany; ^6^Department of Geriatrics, University Medical Center Göttingen, Göttingen, Germany

**Keywords:** developmental stuttering, neurophysiology, speech motor networks, speech and motor deficit, stuttering treatment

Speaking is essential for everyday life: we speak to communicate, sharing our thoughts. However, not everybody speaks easily: speech movements are often out of control in stuttering, and everyday life may be impaired. Developmental Stuttering (DS) is the idiopathic form of the disturbance, usually characterized by speech dysfluencies, such as blocks and repetitions, especially in the initial parts of words and sentences. Also, associated movements (e.g., oro-facial grimaces), accompanying dysfluencies, may be present. DS typically appears in childhood: in the majority of cases, children recover from dysfluencies, but, sometimes, they persist in adulthood. DS is a neurodevelopmental and multifactorial disorder, characterized by the presence of genetic alterations, as well as by abnormalities in the functioning of speech and motor cerebral systems. Neuroimaging/neurophysiological tools have begun to elucidate the dysfunctional neural dynamics of DS: stuttering is seen as a motor/timing disorder related to basal ganglia dysfunction and disconnection of speech-related motor cortical regions. The strong central component of DS influences the functioning of even wider neural networks, such as those related to emotional regulation, also affecting, in interaction with peripheral nervous system, temperamental characteristics, and/or psycholinguistics behaviors. In spite of this amount of knowledge, several questions still remain, which concern, for example, the volitional control of speech or the neural control of motor sequencing/timing (also in relation to response inhibition). These are crucial aspects that need to be considered for a deeper understanding of physiological/pathophysiological bases of DS. Accordingly, the scope of this Research Topic is to help in unraveling the mysteries of (dys)fluency, in stuttering. Hence, we present a collection of original and review contributions around new frontiers in research, trying to contribute to the better understanding of this disorder. Ultimately, 22 articles, including 15 original papers, 4 reviews, 2 hypotheses and theory articles, and 1 brief research report, were produced by 71 of the most influential world-wide experts. The outcome is a potpourri of scientific pieces, tackling a multidisciplinary/integrated vision of DS, to get closer to understanding its mechanisms, and also toward the implementation of more effective evidence-based interventions. Specifically, contributions involve*: (i) Current demographic characteristics of DS; (ii) Causal mechanisms and neural modeling of DS; (iii) Central neurophysiological evidence of (dys)fluency in DS; (iv) Behavioral evidence of motor deficits in DS;* (*v) DS and the peripheral nervous system; (vi) Temperamental and cognitive functioning in DS; (vii) Intervention and rehabilitation in DS*.

## Current Demographic Characteristics of DS

Sommer et al. were able to give a precise and updated picture about the current prevalence and therapy rates of developmental disorders of speech and language, especially in children: they were mainly able to show that stuttering is preferably diagnosed in males, with a prevalence peaking around 5 years of age. Importantly, they demonstrated that the amount of intervention is still not sufficient, especially in childhood. This may have important consequences about the possibilities children may have in learning how to manage their dysfluencies, and thus also about the future invasiveness of stuttering in their lives.

## Causal Mechanisms and Neural Modeling of DS

Alm describes the relationship among speech/motor (frontal) brain regions, which are usually characterized by altered activity in stuttering, and the presence of higher requests of non-oxidative metabolism (i.e., glycolysis), in these same regions. Moreover, the author shows the existence of relations among a series of factors such as genetic abnormalities, lower capacities of using glycolysis, and functional abnormalities of the neural systems of people who stutter (PWS), also explaining that a modulatory (negative) effect may be expected, as a cascade of events, on the functioning of dopaminergic brain systems. Compatibly, Alm extends this view, providing a thorough review about the fundamental role of dopamine in the context of learning, execution, and automatization of movements, especially when considering its relevance for speech: primary mechanisms for the automatization of (complex) motor sequences may result in the “*merging*” of the different parts that are composing the sequence, also involving reinforcement learning processes. In this context, dopamine has an important role, especially when considering the functioning of basal ganglia and cortico-basal-thalamo-cortical mechanisms. Chang and Guenther proposed a model that may help to better explain the neural dynamics related to DS, in the context of a recent and influential model of normal speech production (i.e., the “*Directions into Velocities of Articulators*”-DIVA-model). Specifically, they propose that the primary impairment underlying stuttering may be a dysfunction in the cortico-basal-thalamo-cortical loop, responsible for initiating speech/motor programs. They also analyze three possible loci of impaired neural processing within this system that could lead to dysfluencies: impairments within the basal ganglia, impairments of axonal projections within this network, and impairments in cortical processing of related neural information. As a consequence, a core “*internal*” motor timing deficit in stuttering may be suggested, possibly alleviated by interventions based on the utilization of “*external*” timing cues (e.g., the utilization of a metronome, choral speech etc.). Finally, Alm elegantly analyzed existing relations among some non-genetic contributions in the appearance of DS. The author reports data supporting the proposal that infection with “*group A beta-hemolytic streptococcus*” (GAS) may be a possible (and undiagnosed) cause of stuttering, primarily until the mid-1900s. The proposed mechanism here is an autoimmune reaction, targeting specific neural structures, for example within the basal ganglia system.

## Central Neurophysiological Evidence of (DYS)Fluency in DS

Functional neuroimaging links stuttering to compromised sensorimotor control and deficiencies in auditory-motor integration during speech production. Using fMRI, Sares et al. demonstrated that also vocal pitch compensation may participate in the altered formation of auditory-motor networks in PWS suggesting that, when compared to fluent controls, brain dynamics are notably different when the system is challenged with a mismatch between predicted and actual voice auditory feedback. However, the neural mechanisms underlying these phenomena remain poorly understood, and should be worth studying in the future. Alterations have also been found by Liman et al. that combined motor behavior with the presence of deposits of mesencephalic iron (i.e., an indirect marker of dopaminergic dysfunction), in DS: PWS showed behavioral deficits, such as slower finger tapping, in the presence of enlarged iron deposits on either side of the brain, suggesting that motor deficits in DS may be linked to the presence of a developmental dopaminergic dysfunction that may extend beyond “*classical*” speech functions. Compatibly, Sommer et al. showed that DS resulted in higher amplitudes of motor evoked potentials (MEPs) in hand muscles during spontaneous speech (with respect to fluent speakers), but also in lower MEPs amplitudes during non-verbal oro-facial movements. These findings should be further investigated in the context of exploring MEPs from speech-related muscles, but it can be proposed that speech may request a higher “*neural effort*” in DS (or, on the other hand, that motor inhibitory mechanisms may be altered in PWS, thus affecting complex tasks such as speech execution). Help for the interpretation of these data may arrive from the findings of Vreeswijk et al., which analyzed somatosensory evoked potentials (SEPs) in DS. More specifically, they did not report any difference in SEPs between PWS and fluent speakers, and thus no evidence for dystonia-like sensory overflow of tongue representation in stuttering has been shown. Finally, Jenson et al. reviewed the role of impaired sensorimotor activity in DS (in this case, represented by EEG *mu* rhythm activity, which is generally related to premotor and motor cortical activations), that may be sensitive to basal ganglia inhibitory signaling, sensorimotor feedback, timing and function (also in combination with “*cognitive*”-i.e., working memory-data). They demonstrated that *mu* rhythms might be useful to represent (with high temporal precision) sensorimotor and basal ganglia neural deficits associated with stuttering, thus easily resulting in “*sensory-to-motor*” or “*motor-to-sensory*” alterations of the functional communication among brain regions. In the end, this should be evident in the context of altered motor implementation and/or altered sensorial gating, in DS. Moreover, the authors suggested the possibility that stuttering may also be accompanied by some deficits in more “*cognitive*” and executive functions.

In DS, valuable insights may also be obtained when considering neural dynamics of fluency. In this context, Sengupta et al. showed that, compared to controls, fluent speech preparation in PWS is characterized by a decrease in *theta-gamma* phase coherence with a corresponding increase in *theta-beta* coherence. Higher spectral powers in the *beta* and *gamma* bands were also observed before fluent sentences of PWS. Thus, an altered neural communication during speech planning may be evident in DS, providing evidence for atypical utilization of feed-forward control by PWS, even before fluent speech. In DS, fluency may be temporarily obtained by using techniques such as altered auditory feedback: compatibly, Toyomura et al. showed that delayed auditory feedback (DAF) might allow to better understand the presence of altered brain dynamics in DS, especially when considering auditory suppression induced by own speech, as typically evident in fluent speakers. In DS, a significant suppression was observed when using a 200 ms DAF, with more severe stuttering showing greater speech-induced suppression. These findings are compatible with recent suggestions about the presence of “*delayed*” exchange of neural information in PWS.

## Behavioral Evidence of Motor Deficits in DS

Altered sensorimotor neural dynamics in DS may easily result in abnormalities at a behavioral level: Toyomura et al., starting from the assumption of the presence of an “*internal*” motor timing deficit in DS, speculated that also more general motor behaviors (other than speech) should be disrupted in stuttering. They investigated complex bimanual tasks in PWS and fluent controls, showing that the former performed worse on tasks such as tapping task (but not on bimanual rotation tasks), suggesting that DS may be specifically associated with deficits of timing control, also for general motor behaviors. Compatibly, Korzeczek et al. could not report differences in motor learning capabilities, consolidation and generalization of simple motor sequences of PWS. When considering speech capabilities, Verdurand et al. investigated characteristics of co-articulation in DS: authors showed that, in normal conditions, the co-articulation degree observed in the fluent speech of PWS is lower than fluent speakers. This was also more evident during altered auditory feedback conditions, thus suggesting that larger articulatory movements (and hence, lower levels of co-articulation) could help PWS in the stabilization/compensation of their speech/motor system, further supporting the proposal that stuttering may arise from impaired feed-forward control (trying to use feedback-based motor control for compensation).

## DS and the Peripheral Nervous System

DS may also result in impairments of the peripheral nervous system. In this context, Gattie et al. start from the concept that the larynx's vibrational energy during speech can deflect vestibular mechanoreceptors in humans, and decided to measure vestibular-evoked myogenic potentials in DS. Potential amplitude was smaller in PWS, when compared to fluent speakers. The authors suggest that this finding, interpreted in the context of speech/motor functions, supports the hypothesis regarding the presence of impaired timing networks in DS (as a consequence, additional sensorial/external cues may help regain fluency). Moving toward the autonomic nervous system functions, Walsh et al. examined the relationships among physiological measures of sympathetic arousal, temperament, and communication attitude behavioral indices in children who stutter (CWS) and fluent peers. There was no correlation between sympathetic arousal and stuttering severity or temperament, and for fluent utterances, phasic arousal indices were similar between groups. However, general arousal levels were higher in CWS than controls, independent of whether they performed speech or non-speech tasks. This finding may contrast with increased phasic sympathetic arousal measures available in the literature, and obtained during stuttered speech, thus indicating that actual stuttering may influence the dynamics of the autonomic nervous system. To date, only a few studies have started elucidating ongoing interactions among affective/emotional and speech/motor processes, a perspective that will likely shape future research in the field. In this vein, Tumanova et al. reported that, during challenging picture viewing conditions, CWS showed significantly higher heart rates and a lower respiratory sinus arrhythmia than fluent peers, suggesting that CWS tended to be more emotionally reactive, also employing higher levels of emotional regulation. Emotional reactions and regulatory skills may be critical for the success of DS treatments, especially in childhood.

## Temperamental and Cognitive Functioning in DS

Finally, DS may also result in altered temperament characteristics and/or impaired executive functions, especially in children: Rocha et al. showed that CWS may result in higher impulsivity, emotional reactivity, anger, frustration, and sadness with respect to fluent children. Moreover, they resulted in lower scorings in attention tasks, perceptual sensitivity, reactivity to stressful situations, and tasks measuring executive functioning. Findings indicate that, in CWS, temperament and executive functioning abilities should be taken into account when considering the contribution to the development and/or maintenance of stuttering.

## Intervention and Rehabilitation in DS

All this information should be useful to improve available interventions for DS, also trying to implement new and more effective treatments. In this context, Maguire et al. realized a thorough revision of available evidence, concentrating on the pharmacological options currently available to help clinicians in managing DS. The authors highlight that drugs altering dopamine transmission (e.g., dopamine antagonists) may be the most effective in reducing stuttering severity. They also describe recent possibilities that are currently being investigated (e.g., ecopipam), giving encouraging results. This vision is confirmed by another work of the same group (Maguire et al.) where authors reported that risperidone (an anti-dopaminergic drug) was helpful in augment glucose uptake and metabolism in specific regions that are functionally impaired in the neural system of PWS, such as the left striatum and the Broca's region. Authors also propose that, thanks to risperidone, elevated dopamine activity and striatal hypometabolism of DS may be in part counteracted by mechanisms that may involve striatal astrocytes. Finally, Busan et al. reviewed the current findings and available options for the utilization of non-invasive brain stimulation and neuromodulation in the context of DS, useful to obtain better outcomes in speech fluency and in the functioning of specific “*neural markers*” of the disturbance (such as impairments of speech/motor systems and cortico-basal-thalamo-cortical networks), thus suggesting new and future pathways for research.

## Conclusions

In conclusion, this Research Topic is a unique collection of articles that improve our comprehension of the causal mechanisms and neural dynamics of stuttering. A unifying framework of this multifaceted disorder is beginning to emerge. It will direct and elicit suggestions for future treatments and interventions that will be ultimately useful to overcome this, often under-estimated, speech/motor disturbance.

## Author Contributions

PB wrote the first draft of this editorial. NEN, MRV, PPB, and MS contributed to editing and revising all successive and final versions. As a consequence, all authors have made a substantial, direct, and intellectual contribution to the work, and approved it for publication.

## Funding

This work was supported by the Italian Ministry of Health (Project Code: GR-2018-12366027; grant to PB). The funding sources had no involvement in study design, in the collection, analysis and interpretation of data, in the writing of the report, and in the decision to submit the article for publication.

## Conflict of Interest

The authors declare that the research was conducted in the absence of any commercial or financial relationships that could be construed as a potential conflict of interest.

## Publisher's Note

All claims expressed in this article are solely those of the authors and do not necessarily represent those of their affiliated organizations, or those of the publisher, the editors and the reviewers. Any product that may be evaluated in this article, or claim that may be made by its manufacturer, is not guaranteed or endorsed by the publisher.

